# Population pharmacokinetics and pharmacogenetics of once daily tacrolimus formulation in stable liver transplant recipients

**DOI:** 10.1007/s00228-015-1963-3

**Published:** 2015-10-31

**Authors:** D. J. A. R Moes, S. A. S van der Bent, J. J. Swen, T. van der Straaten, A. Inderson, E. Olofsen, H. W. Verspaget, H. J. Guchelaar, J. den Hartigh, B. van Hoek

**Affiliations:** Department of Clinical Pharmacy and Toxicology, Leiden University Medical Center, Albinusdreef 2, 2333, ZA, Leiden, The Netherlands; Department of Gastroenterology and Hepatology, Leiden University Medical Center, Leiden, The Netherlands; Department of Anesthesiology, Leiden University Medical Center, Leiden, The Netherlands

**Keywords:** Once daily tacrolimus, Advagraf, Population pharmacokinetics, Pharmacogenetics, Liver transplantation

## Abstract

**Purpose:**

The once daily formulation of tacrolimus is an important immunosuppressive drug. Interpatient variability in metabolism has been related to genetic variation in *CYP3A4* and *CYP3A5*. However, in liver transplantation, both donor and recipient genotypes may affect pharmacokinetics. The primary objective of this study was to investigate the effect of CYP3A4*22 and CYP3A5*3 of both donor and recipient on once daily tacrolimus pharmacokinetics. The secondary objective was to develop a limited sampling model able to accurately predict exposure.

**Methods:**

Stable liver transplant patients receiving once daily tacrolimus (*N* = 66) were included. Population pharmacokinetic analysis was performed with patients of whom DNA was available (*N* = 49), and demographic factors, CYP3A4*22 and CYP3A5*3, were tested as covariates. Moreover, a limited sampling model was developed using data of 66 patients.

**Results:**

Pharmacokinetics was best described by a two-compartment model with delayed absorption. CYP3A5*1 carrying recipients engrafted with a CYP3A5*1 carrying liver had an average 1.7-fold higher clearance compared to non-carriers. CYP3A5*1 carrying recipients engrafted with a CYP3A5*1 non-carrying liver or vice versa showed an average 1.3-fold higher clearance compared with non-carriers. CYP3A4*22 was not significantly associated with once daily tacrolimus pharmacokinetics. Using 0, 2, and 3 h postdose as limited sampling model resulted in significantly improved prediction of tacrolimus exposure compared with trough concentration.

**Conclusions:**

Both donor and recipient *CYP3A5* genotype significantly influences tacrolimus once daily pharmacokinetics. In contrast, CYP3A4*22 appears not suitable as biomarker. The developed limited sampling model can be used to accurately estimate tacrolimus once daily exposure.

**Electronic supplementary material:**

The online version of this article (doi:10.1007/s00228-015-1963-3) contains supplementary material, which is available to authorized users.

## Introduction

Prolonged release tacrolimus (Advagraf®) is currently in many centers for the standard formulation of the calcineurin inhibitor tacrolimus in liver transplantation. Advagraf is a once-daily formulation of tacrolimus (ODTac), originally developed to improve adherence which is an important risk factor for rejection and graft loss [1]. Tacrolimus is characterized by a narrow therapeutic window and highly variable pharmacokinetics necessitating therapeutic drug monitoring (TDM) to individualize the dose and prevent rejection or toxicity such as leukopenia and renal toxicity [2]. Tacrolimus is primarily metabolized by the cytochrome P450 enzymes CYP3A4 and CYP3A5 [3]. Differences in activity of metabolizing enzymes are responsible for a large part of the variability in pharmacokinetics [3]. Genetic polymorphisms in *CYP3A4* and *CYP3A5* are known to cause clinically relevant variability in tacrolimus pharmacokinetics in solid organs transplantation [4]. However, since CYP3A4 and CYP3A5 enzymes are both expressed in liver and intestine, in liver transplantation, both genetics of the donor and recipient are of importance. Several studies investigated the role of genetic variants encoding for CYP3A5 in tacrolimus pharmacokinetics in liver transplant recipients [5–11] but were primarily conducted in pediatric and Asian populations. Both donor and recipient CYP3A5 genotype influenced tacrolimus pharmacokinetics in these studies. CYP3A4*22 was only investigated in two different studies in pediatric and Asian liver transplant recipients [6, 12]. Tacrolimus is also a substrate of P-glycoprotein (ABCB1); however, to date, no clinically relevant polymorphisms have been discovered [13, 14] and therefore ABCB1 polymorphisms are not included in the scope of the current study. TDM of ODTac is generally performed using trough concentrations (C_trough_). However, in theory, most informative for true exposure is the area under the blood concentration versus time curve (AUC). This choice has a practical aspect since TDM based on trapezoidal AUC is more laborious for the clinic and inconvenient for the patient since multiple concentration markers are needed for accurate AUC calculation. A limited sampling strategy could help influence the choice of performing TDM based on C_trough_ or AUC. Limited sampling models have been developed for twice-daily tacrolimus [15] in liver transplant recipients and for ODTac in renal transplant recipients [16]; however, whether these are also applicable for ODTac in liver transplant recipients is unknown. The primary objective of this study was to develop a population pharmacokinetic model of ODTac in stable liver transplant recipients and to evaluate the effect of CYP3A5*3 and CYP3A4*22 of both donor and recipient on tacrolimus pharmacokinetics for initial dose differentiation. The secondary objective was to develop a limited sampling strategy to enable prediction of ODTac exposure in liver transplant recipients in an efficient way and to compare it with widely used C_trough_ monitoring.

## Methods

### Patients

During a prospective study, clinical data were collected from 66 stable liver transplant recipients treated with immunosuppressive therapy based on once-daily tacrolimus (Advagraf®, Astellas, Leiden, The Netherlands, further referred to as ODTac) after recent conversion from twice-daily tacrolimus (Prograft®). The DNA of recipient and donor was available for 49 patients. These 49 patients were included for the development of the population PK model and covariate analysis. The donor DNA was not available from the remaining 17 patients. Inclusion criteria of the subjects were at least 18 years old, stable daily dose of twice-daily tacrolimus for at least 3 months, no infections or other complications, bilirubin and albumin levels within clinical reference range, and stable graft function at the moment of conversion. The study was approved by the Medical Ethics Committee of Leiden University Medical Center and patients gave written informed consent. ODTac therapy was started at the same daily dose as of twice-daily tacrolimus. Routine TDM samples were obtained (at least) 2 weeks after conversion from twice-daily tacrolimus to ODTac.

### Bioanalytics

TDM during the study was performed on the basis of trapezoidal rule (kinfit MW/Pharm®), blood concentration at *t* = 0, 1, 2, 3, 4, and 6 h using MW/Pharm version 3.5 (Mediware, Groningen, The Netherlands) [17]. Quantification of tacrolimus TDM samples in whole blood with LC-MS/MS was performed with a validated assay capable of analyzing everolimus, sirolimus, cyclosporine, and tacrolimus simultaneously. Details concerning the LC-MS/MS system are provided in Supplementary File 1. The lower limit of quantification for tacrolimus was 0.4 μg/L. Assay performance, in terms of limits of quantification, was in agreement with the guidelines regarding bioanalytical method validation of Shah et al*.* [18]*.* Supplementary Table 1 shows the samples distribution.

### Genotyping assays

DNA was isolated from EDTA blood from liver transplant recipients and from donor spleen or liver [19]. CYP3A4*22 was determined with TaqMan 7500 (Applied Biosystems, Nieuwerkerk aan de IJssel, The Netherlands) with a custom designed assay, according to the manufacturers’ protocol. CYP3A5*3 was determined with Pyrosequencer 96MA (Isogen, IJsselstein, The Netherlands). Further details with regard to the genotyping protocol are provided in Supplementary Table 2. All allele frequency distributions were in Hardy–Weinberg equilibrium. To explore the combined effect of both recipient and donor genotypes, the following combinations were made for *CYP3A5*: C1, donor and recipient are CYP3A5*1 non-carriers; C2, recipient is CYP3A5*1 carrier and donor is non-carrier; C3, recipient is CYP3A5*1 non-carrier and donor is carrier; and C4, both donor and recipient are CYP3A5*1 carriers. The following combinations were made for *CYP3A4*: C1, donor and recipient are CYP3A4*22 non-carriers; C2, recipient is CYP3A4*22 carrier and donor is non-carrier; C3, recipient is CYP3A4*22 non-carrier and donor is carrier; and C4, both donor and recipient are CYP3A4*22 carriers.

### Pharmacokinetic modeling

Nonlinear mixed effects modeling was used to estimate once-daily tacrolimus (Advagraf) pharmacokinetic parameters from blood concentration-time data. NONMEM (v7.2.1, Icon Development Solutions, Ellicott City, MD) was used for modeling ODTac pharmacokinetics, using PsN toolkit 3.7.6 [20] and Piranã version 2.8.1 [21] as modeling environment. Results were analyzed using the statistical software package R (v2.15.2). First-order conditional estimation method with interaction (FOCE-I) was used throughout the analysis. Model selection was based on statistical significance, goodness of fit, and stability. Throughout the model building process, an altered model was chosen over a precursor model if a difference in the objective functions (−2 log-likelihood) was >6.63 (*P* < 0.01, with 1 degree of freedom, assuming an *X*^2^ distribution).

### Base model

Initially, the model was developed exclusively on pharmacokinetic data without covariates. The concentration-time data were reviewed for completeness and consistency of sampling and dosing times. Plots of observed concentration-time data were examined. Subsequently, one- and two-compartmental pharmacokinetic models with first-order elimination were compared to find the best fit of the concentration-time data. The value for bioavailability was fixed to 0.23 which was based on literature [22]. Furthermore, the use of transit compartments and a lag time for drug absorption were explored.

### Covariate analysis

Diagnostic plots were constructed of the random effects of clearance (CL), distribution volume of the central compartment (Vc) and absorption rate constant (*K*_a_) versus the recipient demographic (age, weight, sex, ethnicity, height, lean body weight (LBW), ideal body weight (IBW), body surface area (BSA), body mass index (BMI), hematocrit, hemoglobin, albumin, creatinine, primary diagnosis and co-medications (also weighted residual vs. co-medications plots), and donor and recipient pharmacogenetic (*CYP3A4 and CYP3A5* polymorphisms) characteristics. Criteria for evaluation of co-medication were a minimum frequency of administration and probability of interaction based on literature. Genetic polymorphisms were selected based on theoretical relationship and minimal allele frequency (>0.10) to assure detection of clinically relevant effects on ODTac PK. Based on these plots, further testing in the pharmacostatistical model was performed. Subsequently, selected covariate relationships were evaluated by forward inclusion and backward deletion procedure (*P* < 0.05 and *P* < 0.01, respectively). A covariate effect was only maintained in the model if the inclusion resulted in a reduction in random variability and improved model fit.

The influence of continuous covariates on pharmacokinetic parameters was tested according to an allometric function. For example, the effect of ideal body weight on apparent clearance (CL/F) was tested using the following equation:

$$ \mathrm{C}\mathrm{L}/\mathrm{F}=\mathrm{T}\mathrm{V}\left(\mathrm{C}\mathrm{L}\right)\times {\left(\mathrm{I}\mathrm{B}\mathrm{W}/\mathrm{mediancov}\right)}^{\theta_{\mathrm{IBW}}} $$

where TV(CL) is the typical value of clearance for a patient with the median covariate value (mediancov) and θ_IBW_ is the estimated influential factor for ideal body weight. The effect of the genetic polymorphisms and other categorical covariates was tested using the equation:

$$ CL/\mathrm{F}=TV(CL)\times \left(1+{\theta}_{cov}\right) $$

where TV(CL) represents the clearance of patients with *θ*_cov_ equal to 0 (i.e., CYP3A5*3/*3 carriers or CYP3A4*1/*1) and *θ*_cov_ is the estimated influential factor for the comparator group. For instance, if the estimated value *θ*_cov_ is 0.3, the clearance of the mutant group is on average 30 % higher than the reference group. Covariates were tested for all the pharmacokinetic parameters for which interpatient variability was estimated.

### Visual predictive check with prediction-correction

A prediction corrected visual predictive check (predcorrVPC) was used to evaluate the performance of candidate and final models of ODTac pharmacokinetics, by simulation of 500 simulated datasets [23]. Bin separators in the VPC were set at the lowest densities of sample points over time, since observations were spread around nominal time points, i.e., this positions the bins such that the periods with densest sampling were in the middle of the bins. Shrinkage in between subject variability (BSV) and residual errors was automatically calculated by NONMEM v7.2.1. to assess the informativeness of the data for using individual predictions in the evaluation of model fit. The distribution (median and 10th and 90th percentiles) of the simulated concentration-time courses was compared with the distribution of the observed values in the original dataset. Differences and overlap of the simulated and original distributions indicated the accuracy of the identified model.

### Limited sampling strategy

#### Patients and data collection

For the development of a limited sampling strategy, 66 AUCs from 66 different patients were available, consisting of the 49 patients used in covariate analysis and an additional 17 patients of whom no DNA was available. Demographic parameters of these 66 patients are presented in Table 1. Pharmacokinetic profiles consisted of six blood samples collected over 6 h (before dose and 1, 2, 3, 4, and 6 h postdose).

**Table 1:** Clinical characteristics

Population pharmacokinetics and pharmacogenetics model dataset				Limited sampling dataset		
Recipient characteristics	Number (proportions)	Mean ± SD	Median (range)	Number (proportions)	Mean ± SD	Median (range)
Male	31 (63 %)			41 (62 %)		
Female	18 (37 %)			25 (38 %)		
Age (years)		54 ± 11	55 (29–69)		54 ± 11	55 (29–69)
Caucasian	45 (92 %)			59 (89 %)		
Weight (kg)		84 ± 18	84 (50–131)		83 ± 17	82 (50–131)
Body Surface Area(m^2^)		2 ± 0.23	2 (1.5–2.6)		2.0 ± 0.23	2.0 (1.5–2.6)
Lean Body Mass (kg)		59 ± 10	59 (40–80)		59 ± 9.8	59 (40–80)
Ideal Body Weight (kg)		66 ± 7	68 (51–80)		66 ± 7.5	68 (51–82)
Height (cm)		173 ± 8	174 (155–190)		173 ± 9	174 (155–193)
Creatinine (μmol/L)		95 ± 26	97 (41–191)		97 ± 27	96 (41–191)
Albumin (g/L)		45 ± 3	45 (35–54)		45 ± 3	45 (35–54)
Hemoglobin (mmol/L)		8.48 ± 0.99	8.6 (6–10.2)		8.5 ± 1.0	8.6 (5.7–10.8)
Hematocrit (L/L)		0.42 ± 0.04	0.42 (0.30–0.50)		0.42 ± 0.04	0.42 (0.29–0.52)
Primary diagnosis						
Alcoholic liver disease	12 (24.5 %)			16 (24 %)		
Hepatitis B	1 (2 %)			5 (8 %)		
Hepatitis C	4 (8 %)			6 (9 %)		
Primary sclerosing cholangitis	9 (18 %)			10 (15 %)		
Primary biliary cirrhosis	2 (4 %)			2 (3 %)		
Nonalcoholic steatohepatitis	3 (6 %)			3 (5 %)		
Wilson’s disease	1 (2 %)			3 (5 %)		
Cystic liver disease	4 (8 %)			5 (8 %)		
Hepatocellular carcinoma	1 (2 %)			1 (2 %)		
Cryptogenic liver disease	2 (4 %)			2 (3 %)		
Auto-immune hepatitis	1 (2 %)			1 (2 %)		
Other	9 (18 %)			12 (18 %)		
Exposure						
Advagraf dose (mg)		3.6 ± 2.2	3 (0.5–14)		3.5 ± 2.0	3 (1–14)
Advagraf AUC_24_ (μg*h/L)		170 ± 55	162 (72–330)		164 ± 53	162 (50–330)
Concentrations (μg/L)		8.5 ± 3.7	8.1 (1.8–20.7)		8.3 ± 3.7	8 (1.3–20.7)
Renal function						
Creatinine clearance (mL/min)^a^		87 ± 24	87 (33–120)		85 ± 23	85 (33–120)

^a^ MDRD when <60 mL/min, Cockgroft Gault when >60 ml/min (cutoff 120 ml/min), AUC_24_ = area under the blood concentration-time curve (24 h)

#### Pharmacokinetic and statistical analysis

For the development of a limited sampling model (LSM), we calculated the predictive performance of different limited sampling methods (limited sampling models and a limited sampling formula). “True” exposure (FULL AUC_24_) was calculated using post hoc estimation in NONMEM with the final model (AUC_24_ = ((DOSE*F)/CL). LSM AUC_24_ was calculated by selecting several concentration-time points and combinations of time points and fitting the data points with post hoc estimation in NONMEM with the final model. Subsequently, the FULL AUC_24_ and LSM AUC_24_ of all individuals were compared. Since C_trough_ is widely used in TDM also, a limited sampling formula (LSF) by means of a linear regression equation of C_trough_ vs. FULL AUC_24_ was calculated to show the difference between the limited sampling formula and a limited sampling model. Pearson correlation coefficient test was performed to determine the correlation between FULL AUC_24_ and limited sampling method AUC_24_. To evaluate the performance of the limited sampling methods, the 17 additional patients were also evaluated separately and compared with the overall results. The formulas of the used predictive performance measures are presented in Supplementary Table 3.

## Results

### Patients, pharmacokinetic, and pharmacogenetic data

Forty-nine adult liver transplant recipients, 31 men, and 18 women were included for the development of the population PK model and covariate analysis. The majority was Caucasian (92 %). Mean age was 54 ± 11 years (range, 29–69 years). Mean bodyweight was 77.5 ± 11.8 kg (50–121 kg). The dataset consisted of 282 samples. Demographic characteristics and details about the distribution of dosage and exposure are found in Table 1. Genotype distributions for CYP3A5*3 and CYP3A4*22 are presented in Table 2.

**Table 2:** Genotype frequencies in studied population (*n* = 49)

Gene	SNP(s)	Nucleotide position and alleles	Genotype	Frequency [N (%)]
Recipient				
CYP3A4				
	rs35599367	C > T	C/C	36
			C/T	11
			T/T	2
CYP3A5				
	rs776746	6986A > G	A/A	36
			G/A	10
			G/G	3
Donor				
CYP3A4				
	rs35599367	C > T	C/C	40
			C/T	8
			T/T	1
CYP3A5				
	rs776746	6986A > G	A/A	40
			G/A	9
			G/G	0
Combination				
CYP3A4				
			C1	30
			C2	10
			C3	6
			C4	3
CYP3A5				
			C1	32
			C2	8
			C3	4
			C4	5

CYP3A5: C1 donor and recipient are CYP3A5*1 non-carriers; C2, recipient is CYP3A5*1 carrier and donor is non-carrier; C3, recipient is CYP3A5*1 non-carrier and donor is carrier; and C4, both donor and recipient are CYP3A5*1 carriers. For CYP3A4, C1 donor and recipient are CYP3A4*22 non-carriers; C2, Recipient is CYP3A4*22 carrier and donor is non-carrier; C3, Recipient is CYP3A4*22 non-carrier and donor is carrier; and C4 both donor and recipient are CYP3A4*22 carriers.

### Structural model development

**Fig. 1:**
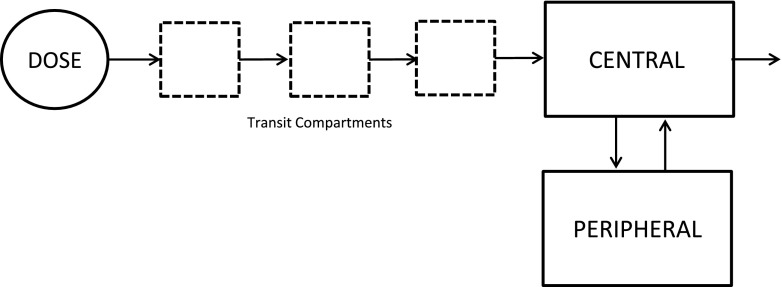
Schematic representation of the linear two-compartment model with first-order absorption and elimination of once daily tacrolimus (Advagraf) including the transit compartments to describe the absorption phase

The pharmacokinetic data of once-daily tacrolimus was best described by a two-compartmental model with delayed first-order absorption and first-order elimination from the central compartment. The delayed absorption of once daily tacrolimus was best described with three transit compartments: a first-order rate constant describing the transfer from the dose compartment into the transit compartments and finally into the central compartment (Fig. 1). Random effect parameters for interindividual variability in clearance (CL), volume of central compartment (V_c_), and rate of absorption (K_a_) were identified. Thereafter, the random effects were tested for structural relationship with dose and time to create a model with unbiased and randomly distributed random effects for covariate analysis. The shrinkage for random effect parameters on CL/F (0 %) V_c_/F (10 %) and K_a_ (15 %) was small which supports unbiased covariate inclusion of the final model. The structural pharmacokinetic model indicated an apparent clearance (CL/F) of 4.77 L/h, an apparent central distribution volume of 87.3 L (V_c_/F) and an apparent peripheral distribution volume of 142 L. The absorption rate constant was 3.65 h^−1^. Intercompartmental clearance was 14.1 L/h. The pharmacokinetic data showed interindividual variability in CL/F of 45.4 % with a range of 1.17–17.2 L/h.

### Covariate analysis

#### Demographics

The base model was used for the demographic and genetic covariate analysis. Diagnostic plots of random effects of the pharmacokinetic parameters in the initial model against age, weight, sex, hematocrit, hemoglobin, albumin, height, creatinine, IBW, BSA, BMI, LBW, co-medication, primary diagnosis, and ethnicity were built. Plots of weighted residuals versus co-medications were also constructed in case there were changes in concurrent medication regimens. The evaluated co-medications can be found in Supplementary Table 4. Only IBW and height showed a significant relationship in the univariate covariate analysis (*P* < 0.05), however, in the multivariate analysis (*P* < 0.01), these covariates were not significant. The following were not significant covariates on CL/F, V_c_/F, or K_a_: age, weight, sex, hematocrit, hemoglobin, albumin, creatinine, BSA, BMI, LBW, co-medication, primary diagnosis, and ethnicity.

#### Pharmacogenetics

**Fig. 2:**
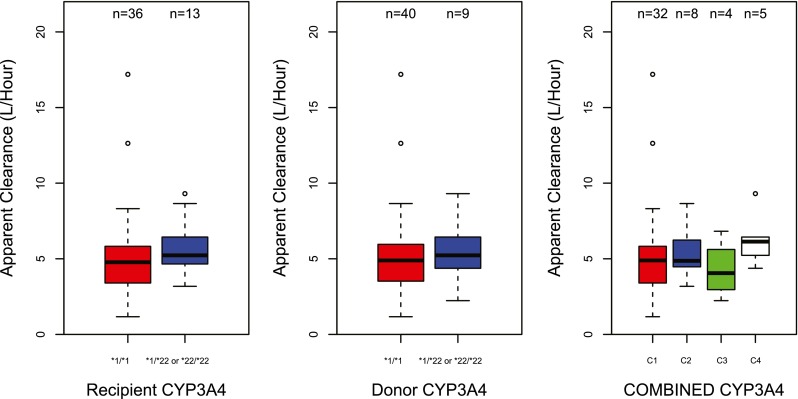
Boxplots representing the average once-daily tacrolimus apparent clearance (L/h) of the different genotype groups with error bars and the number of patients in each group. CYP3A4 (*1/*1 = CYP3A4*22 non-carriers, *1/*22 or *22/*22 = CYP3A4*22 carriers. CYP3A4 combination: C1 donor and recipient are CYP3A4*22 non-carriers; C,: recipient is CYP3A4*22 carrier and donor is non-carrier; C3, recipient is CYP3A4*22 non-carrier and donor is carrier; and C4, both donor and recipient are CYP3A4*22 carriers. *significant

**Fig. 3:**
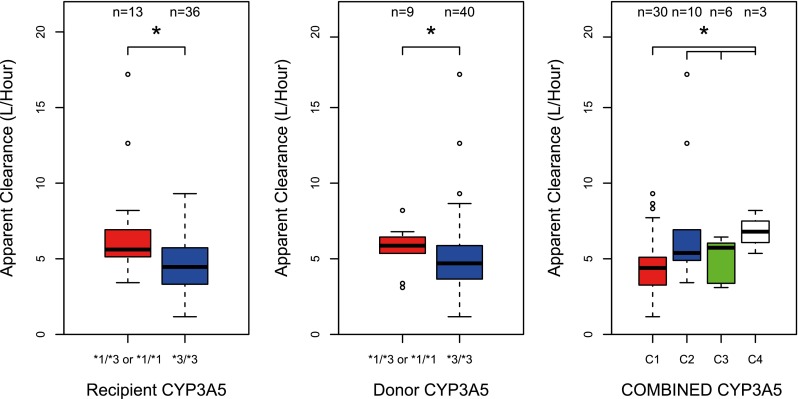
Boxplots representing the average once-daily tacrolimus apparent clearance (L/h) of the different genotype groups with error bars and the number of patients in each group. CYP3A5 (*1/*3 or *1/*1 = CYP3A5*1 carriers, *3/*3 = CYP3A5*1 non-carriers), CYP3A5 combination: C1 donor and recipient are CYP3A5*1 non-carriers; C2, recipient is CYP3A5*1 carrier and donor is non-carrier; C3, recipient is CYP3A5*1 non-carrier and donor is carrier; and C4, both donor and recipient are CYP3A5*1 carriers. *Significant

Diagnostic plots were created of random effects of CL, V_c_, and K_a_ against genetic polymorphisms in *CYP3A4 and CYP3A5*. The summary of the results of the effect of CYP3A4*22 and CYP3A5*3 on tacrolimus clearance is presented in Table 3 and graphically shown in Figs. 2 and 3. CYP3A4*22 was not significantly associated with tacrolimus CL/F. In contrast, CYP3A5*3 showed a significant effect (*P* < 0.05). Recipients with a genotype with at least one increased activity allele had an average 38 % higher clearance compared to non-carriers. Patients with a donor liver carrying at least one increased activity allele had an average 38 % higher clearance compared to non-carriers. Furthermore, when combining both donor and recipients genotype, C2, C3, and C4 showed higher clearance compared to C1 (33, 33, and 71 %, respectively) (*P* < 0.01). The population pharmacokinetic parameters obtained with the base and final model are presented in Table 4.

**Table 3:** CYP3A4 & CYP3A5 covariate analysis results

Covariate tested	ΔOFV	*P* value		Mean value (%)	95 % CI
Advagraf base model					
+ Recipient CYP3A4*22	1.330	0.249		17	−9 to 43
+ Donor CYP3A4*22	0.391	0.532		0	−29 to 28
+ CYP3A4*22 combination	2.036	0.565	C1	0	−16 to 16
			C2	12	−17 to 41
			C3	−16	−50 to 18
			C4	19	−19 to 56
+ Recipient CYP3A5*3	5.551	*0.018*		38	6 to 70
+ Donor CYP3A5*3	4.54	*0.033*		38	5 to 71
+ CYP3A5*3 combination	9.106	*0.003*	C1	0	−15 to 15
			C2	33	−4 to 71
			C3	33	−8 to 74
			C4	71	31 to 110

∆*OFV*(delta Objective Function Value) >3.84 (*P* < 0.05) and >*6.64* (*P* < 0.01, chi-square test), Mean Value = the percentage deviation compared to the reference group*.*

**Table 4:** Summary of population pharmacokinetic parameter estimates from the base and final model with relative standard error and shrinkage (%) and parameter estimates from 1000 bootstrap replicates with 95 % CI

	Base model			Final model			1000 bootstrap runs	
PK parameter	Mean value	RSE(%)	Shrinkage (%)	Mean value	RSE(%)	Shrinkage (%)	Median value	95 % CI
CL (L/h)	4.77	7		4.21	8		4.22	3.58 to 4.97
F (fixed)	0.23	–		0.23	–		0.23	–
V_c_ (L)	87.3	16		88.3	12		82.2	56.6 to 110.8
Q (L/h)	14.1	20		14	22		14.8	11.0 to 26.5
V_p_ (L)	142	28		145	41		131.5	86.8 to 348.4
K_a_ (h-1)	3.65	10		3.76	10		3.61	2.81 to 4.67
Cyp3A5*3 on CL								
C1 (Reference group)(%)				0			0	−15 to 15
C2 (%)				33			32.3	−2.1 to 81.4
C3 (%)				33			30.5	−7.0 to 90.0
C4 (%)				71			67.7	35.1 to 121.3
Interindividual variability								
IIV CL (CV%)	45.4	14	0	42.8	13	0	41.6	31.3 to 53.9
IIV Vc (CV%)	86.2	14	10	86.3	14	9	87.3	63.6 to 133.1
IIV Ka (CV%)	67.4	15	16	65.9	14	15	64.9	45.1 to 91.7
Random residual variability								
σ^1^ (proportional error (%))	13	9	23	13	8	23	12.5	10.4 to 14.6

**Fig. 4:**
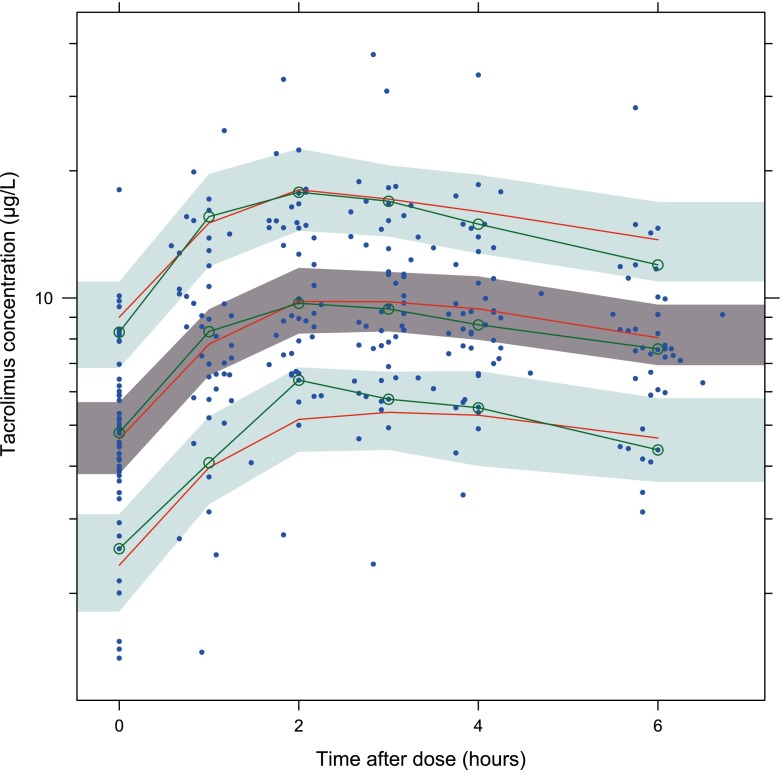
Prediction corrected visual predictive check with 80 % prediction interval. The observed concentrations are shown as *closed circles*. The *lines with round circles* represent the observation intervals. The *solid lines* represent the prediction intervals. The *shaded areas* around the prediction intervals represent the 95 % confidence interval around each of the prediction interval

The evaluation of the precision of the pharmacokinetic parameters was performed with 1000 bootstrap replicates. The percentage of successful runs was 84 %. Moreover, the parameter estimates of the non-successful runs were analyzed and did not deviate from the parameter estimates of the successful runs. The mean values for all fixed effect parameters were within 15 % of those obtained by the final model, indicating good reliability (Table 4). Since different dosages were used during the study, the performance of the model was evaluated with a predictive corrected visual predictive check [15] (Fig. 4). Predictive and observed intervals (10, 90, and median) are almost identical, showing good predictive performance of the final model.

**Fig. 5:**
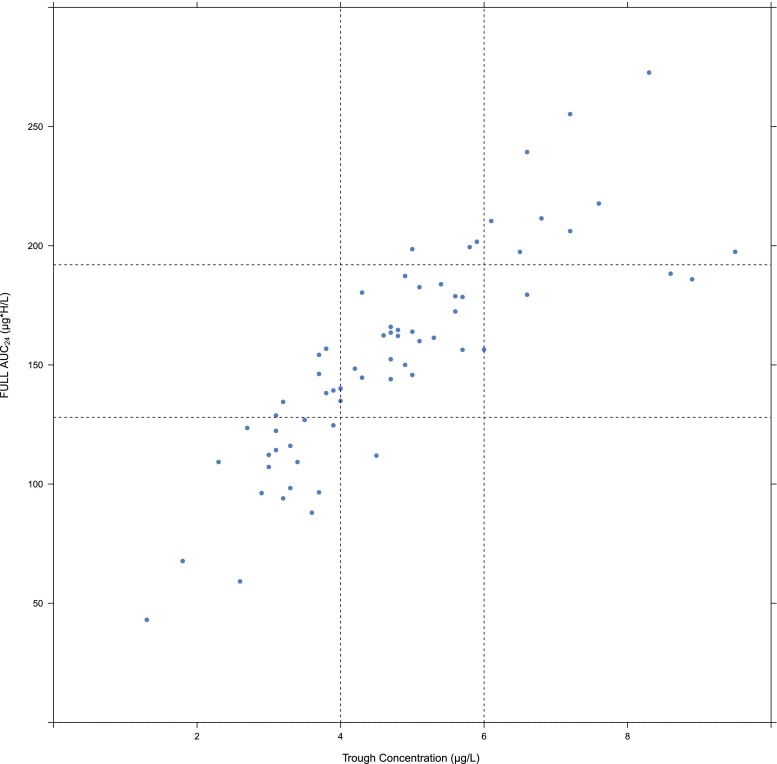
AUC_24_ correlation of PK profiles of 66 different patients (dose range 1–14 mg). *Dotted lines* crossing *x*-axis represent C_trough_ target area. *Dotted lines* crossing the *y*-axis represent the 20 % deviation area from the target AUC_24_ of 160 μg*h/L (128–192 μg*h/L). AUC_24_ = area under de blood concentration-time curve from time zero to 24 h

In Fig. 5, the C_trough_ and FULL AUC_24_ correlation is presented with the C_trough_ and corresponding FULL AUC_24_. Although a relatively good correlation is found between C_trough_ and AUC_24,_ a relatively large amount of patients remains at risk for under or over exposure. The C_trough_ target range (4–6 μg/L) corresponds with a wide AUC_24_ range and vice versa. For instance, an AUC of around 160 μg*h/L (±20 % range, 128–192 μg*h/L) corresponds with a C_trough_ of 3.8 but also with a C_trough_ of 9.5 μg/L.

### Development of limited sampling model

**Fig. 6:**
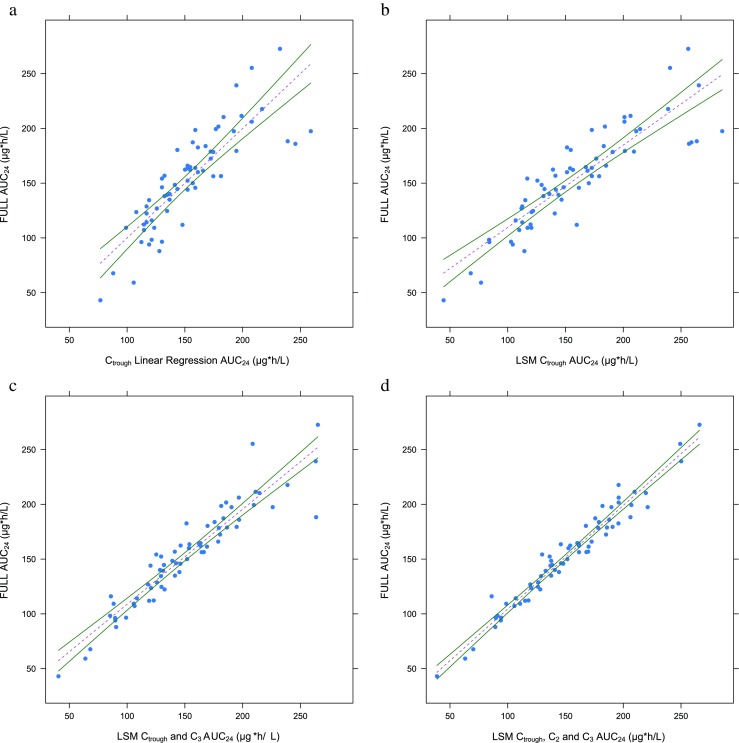
Regression line (*dotted lines*) plots of limited sampling methods with 95 % confidence intervals (*solid lines*). **a** Predictive performance of C_trough_ as limited sampling formula, **b** predictive performance of C_trough_ with as sampling model, **c** predictive performance of C_trough,3_ as limited sampling model, **d** predictive performance of C_trough,2,3_ as limited sampling model

The results of the development of a LSM and the LSF of C_trough_ are shown in Fig. 6 and Supplementary Table 5. Predictive performance measurements used are the percentage of predicted AUC’s within a 15 % range of the “true” AUC, discordance (%) (meaning a predicted AUC leading to incorrect dose change), different ways of describing bias, and imprecision (MPE, MAPE, and RSME) and correlation. Figure 6 shows results of four LSMs, both regression lines with 95 % CI as measurements of predictive performance. The limited sampling formula of C_trough_ (22.213*C_trough_ + 47.983) for once-daily tacrolimus in predicting systemic exposure had a moderate correlation with full trapezoidal AUC_24_ (a discordance of 18.2 %, a mean absolute percentage prediction error of 13.3 %, and *R*^2^ = 0.72). The best single point marker was C_trough_ (discordance, 12.1 %; mean absolute percentage prediction error, 11.42 %; *R*^2^ = 0.78). The best two point markers were C_trough_ and C_3_ (discordance, 3.0 %; mean absolute percentage prediction error, 5.2 %; *R*^2^ = 0.88). The best three point markers were C_trough_, C_2_, and C_3_ (a discordance of 1.52 % and a mean absolute percentage prediction error of 7.61 %, and *R*^2^ = 0.97). The widely used C_trough_ showed less performance with LSF and LSM compared to the two point markers C_trough_ and C_3_. These results were confirmed when evaluating the limited sampling models using only the 17 liver transplant recipients which were not used for the development of the population pharmacokinetic model as showed in Supplementary Table 5 below.

## Discussion

The pharmacokinetics of ODTac in stable liver transplant recipients is best described by a two-compartmental model with first-order absorption and lag time. The delayed absorption was best described with three transit compartments. This study shows for the first time that ODTac pharmacokinetics is not significantly influenced by CYP3A4*22. In contrast, *CYP3A5* genotype of both donor and recipient influences ODTac pharmacokinetics to a clinically relevant extent. Our data indicate that both donor and recipient genotype should be considered when establishing an initial ODTac dose of liver transplant recipients. The contribution of the intestine CYP3A5 genotype (recipient) and liver genotype (donor) to tacrolimus pharmacokinetics was comparable. Without considering the genotype, recipients engrafted with a CYP3A5*1 carrying liver could be at risk of tacrolimus underexposure. This is also technically feasible since tacrolimus is often initiated several days posttransplantation, enabling sufficient time to genotype the donor liver. ODTac pharmacokinetics was not significantly influenced by weight, age, sex, hematocrit, hemoglobin, albumin, height, BMI, BSA, LBW, primary diagnosis, co-medication, and ethnicity. Furthermore, the development of a limited sampling model resulted in identification of a three point concentration marker for accurately predicting ODTac exposure.

In this study, the mean apparent clearance and apparent distribution volume of the central compartment of ODTac were 4.77 L/h and 87.3 L, respectively. The PK parameter estimates found in this study are in agreement with those (CL/F = 5.72 L/H) found by Yang et al*.* [25] and Woilard et al*.* (CL/F = 4.6 L/H if F = 0.23) [16] when taking differences in patient population (Asian, pediatric vs. adult Caucasian) and differences in modeling into account.

The relationship between ethnicity and clearance as found in previous studies [26, 27] could not be identified in our study. This is most likely caused by the lack of data on ethnicity of the donor, and the majority (92 %) of the recipients was of Caucasian origin. The fact that we found no effect for concomitant medications is probably caused by the fact that the medications previously found to be of influence on tacrolimus clearance [3] were not administered to our liver transplant recipient population. Prednisolone was administered in too low doses to be of influence (≤10 mg). Although established before [28, 29], hematocrit and hemoglobin were not identified as a significant covariate in this analysis, most likely explained by the relative narrow range within the patient population (0.3–0.5 L/L and 6–10.2 mmol/L, respectively).

This is the first comprehensive study investigating the effect of CYP3A4*22 and CYP3A5*3 of both donor and recipient on ODTac pharmacokinetics in stable adult Caucasian liver transplant recipients. These polymorphisms were studied before in relation to pharmacokinetics of everolimus, tacrolimus, and cyclosporine in renal transplant recipients [30, 31]. For tacrolimus clearance, the relationship to *CYP3A5* genotype has been identified previously [5, 6, 30, 32] and has been adopted in clinical practice in some transplantation centers. However, CYP3A4*22 has shown less conclusive results [30, 33, 34]. In liver transplant recipients, CYP3A4*22 was only investigated in an Asian population where no mutations were identified [12]. The limited effect of CYP3A4*22 is probably also masked by the more dominant effect of CYP3A5*3. Allele frequencies found in our dataset were similar to those published previously [35]. The remaining variability of our final model was 42.8 % (a sum of inter- and intraindividual variability) and reflects the wide interindividual variability in CYP3A4/5 expression [36]. Based on our result, we propose to implement genotyping of both donor and recipient to establish an initial dose for ODTac in liver transplant recipients. When aiming for an AUC_24_ of for instance 320 μg*h/L (C_trough_ 10–12 μg/L), this would mean an initial dose of 6 mg ODTac for non-CYP3A5*1 carrying liver transplant recipients, 7.5 mg ODTac for CYP3A5*1 carrying recipients engrafted with a CYP3A5*1 non-carrying liver or vice versa, and 10 mg ODTac for CYP3A5*1 carrying recipients engrafted with a CYP3A5*1 carrying liver. Future studies should investigate whether genotype-based dosing also leads to improved clinical endpoints such as lower rejection rates and improved graft survival.

In the present study, a large number of concentration-time data was used for the population pharmacokinetic analysis. However, our study has some limitations: Interoccasion variability could not be established since ODTac AUC-measurements were only performed on one occasion. Concentrations up to 6 h were collected and not up to 24 h. Nevertheless, the PK parameters found were in accordance to previously reported ODTac PK studies [6, 16] which did not have these disadvantages. Furthermore, data collected from stable liver transplant recipients were used. In general, pharmacokinetics shortly after transplantation is more variable. Using stable liver transplant recipients for this analysis however results in a more unbiased view on the specific genotype contribution on ODTac pharmacokinetics. However, confirmation of the current findings in unstable liver transplant recipients would strengthen our conclusions. Furthermore, a larger study could help to narrow down the 95 % confidence intervals of the genotype effects.

C_trough_ monitoring of ODTac is globally widely adopted. Besides the higher impact of assay variability when using one marker to predict ODTac systemic exposure, the correlation between C_trough_ and AUC_24_ is not optimal as shown in the results and could theoretically lead to therapy failure when exposure is 20 % higher or lower as intended [37]. Suboptimal predictive performance of a TDM marker can lead to incorrect dose adjustments resulting in exposure outside the target range. Using trapezoidal, AUC_24_ has the disadvantage of requiring a relatively high number of blood samples to reach good predictive performance which is a very invasive and inconvenient way of performing TDM. A good alternative is the use of a LSM with good predictive performance.

Since correlation coefficient can be misleading bias, imprecision was calculated to assess the performance of the different LSMs according to the guidelines proposed by Sheiner and Beal [24]. In our study, C_trough_ monitoring had a worse performance in estimating AUC_24_ when using LSF and LSM as compared with C_trough,2,3_ in LSM. Especially, the LSF resulted in a 27 % higher percentage of patients outside of the 15 % radius of the FULL AUC_24_ and a 16.7 % rise in discordance. C_trough,2,3_ and C_trough,1,3_ showed comparable performance and are both suitable as limited sampling model. These results were confirmed with limited sampling evaluation of 17 patients which were not used for the development of the final model. In summary, using the three point markers C_trough_, C_2_, and C_3_ as limited sampling model is the best option, when taking predictive performance and inconvenience of the sampling for both patient and the clinic into account. The three-point LSM marker C_trough,2,3_ and comparable results of C_trough,2,3_ are in accordance with what previously has been found for ODTac in renal transplant recipients [16].

In conclusion, this study shows that the population pharmacokinetics of ODTac in adult liver transplant recipients is best described by a two-compartment pharmacokinetic model with delayed absorption described by three transit compartments. CYP3A5 genotype but not CYP3A4 genotype of both donor and recipient should be taken into account to establish an initial dose for once-daily tacrolimus. Tacrolimus blood concentrations measured at 0, 2, and 3 h postdose can be used to accurately estimate ODTac systemic exposure, a clear improvement compared to the widely used C_trough_ monitoring.

## Electronic Supplementary Material

Supplementary File 1 (DOCX 13 kb)

Supplementary Table 1 (XLS 32 kb)

Supplementary Table 2 (XLS 30 kb)

Supplementary Table 3 (XLS 34 kb)

Supplementary Table 4 (XLS 25 kb)

Supplementary Table 5 (XLS 43 kb)
