# Supplementary material for: Population pharmacokinetics and pharmacogenetics of once daily tacrolimus formulation in stable liver transplant recipients
Source: Eur J Clin Pharmacol. 2015 Oct 31;72:163–74. doi: 10.1007/s00228-015-1963-3 (PMC4713720; doi:10.1007/s00228-015-1963-3)
Supplement: Supplementary file 1 — (DOCX 13 kb) [file 228_2015_1963_MOESM1_ESM.docx]

Technical details of the HPLC-MS/MS system:

The system consisted of an Ultimate 3000 autosampler, a thermostatted column compartment TCC 100 and a p680 HPLC dual low-pressure gradient pump (analytical). All were purchased from Dionex Benelux B.V (Amsterdam, The Netherlands). The MS/MS used was a Quattro micro™ API Tandem Quadrupole system from Waters corporation, Milford, U.S.A.

200 μL of blood samples, controls or calibrators were diluted with 200 μL 0.1 M ZnSO4 and 500μL internal standard solution. Internal standard solution consisted of 100 μL 16 μg/L ascomycin in methanol and 25 mL acetonitrile (LiChrosolv, Merck KGaA, Darmstadt Germany). A 6+1 multilevel calibrator set (0. 2.35, 5.96, 11.9, 17.6, 24.6, 42.2 μg/L) was used, which was obtained from Chromsystems (Munich, Germany). Blood control levels 1, 2, 3 and 4 (2.6, 7.1, 14.8 and 32.2 μg/L) were obtained from Chromsystems (Munich, Germany). After diluting, 2 minutes of vortex mixing followed by 5 minutes of centrifugation at 13000 rpm was conducted. After centrifugation, the supernatant was transferred into a cylindrical crimp neck autosampler vial.

A 50 μL aliquot of supernatant was injected into an online Solid Phase Extraction column (Cartridge Hysphere 5C18 HD, 7μm particle size 10 x 2 mm, Spark, Emmen, The Netherlands) for sample clean-up and enrichment. For sample elution two mobile phases were used: mobile phase A: 0.1% v/v formic acid + 2 mM ammonium acetate in water and mobile phase B: 0.1% v/v formic acid + 2 mM ammonium acetate in methanol. The elution gradient used on the SPE column was 50% A and 50% B for 2 minutes, followed by 0.8 minute 100% B and 1.5 minutes 50% A and 50% B for elution of tacrolimus and IS for isocratic liquid chromatography on the pre-column (Hypersil 4 x 2 mm, Phenomenex) and analytical column (Hypersil Phenyl 50 x 3 mm, 3μm particle size, Thermo Scientific). The column oven was set at 55°C. The elution gradient for chromatographic separation to the MS was 10% A and 90% B at a flow rate of 600 μL/min. Mass-spectrometric detection was in positive ion mode using selected reactant monitoring (tacrolimus m/z 821.4→768.4, internal standard, ascomycin, m/z 809.4→756.5).
